# Chick Chorioallantoic Membrane Assay: A 3D Animal Model for Study of Human Nasopharyngeal Carcinoma

**DOI:** 10.1371/journal.pone.0130935

**Published:** 2015-06-24

**Authors:** Xue Xiao, Xiaoying Zhou, Huixin Ming, Jinyan Zhang, Guangwu Huang, Zhe Zhang, Ping Li

**Affiliations:** 1 Department of Otolaryngology-Head & Neck Surgery, First Affiliated Hospital of Guangxi Medical University, Nanning, China; 2 Department of Pathology, First Affiliated Hospital of Guangxi Medical University, Nanning, China; Gustave Roussy, FRANCE

## Abstract

Nasopharyngeal carcinoma (NPC) is a highly invasive and metastatic head and neck cancer. However, mechanistic study of the invasion and metastasis of NPC has been hampered by the lack of proper *in vivo* models. We established an *in vivo* chick embryo chorioallantoic membrane (CAM) model to study NPC tumor biology. We found 100% micro-tumor formation 3 days after inoculation with NPC cell lines (4/4) or primary tumor biopsy tissue (35/35). The transplanted NPC micro-tumors grew on CAMs with extracellular matrix interaction and induced angiogenesis. In addition, the CAM model could be used to study the growth of transplanted NPC tumors and also several important steps of metastasis, including tumor invasion by detecting the extent of basement membrane penetration, tumor angiogenesis by analyzing the area of neo-vessels, and tumor metastasis by quantifying tumor cells in distant organs. We established and described a feasible, easy-to-manipulate and reliable CAM model for *in vivo* study of NPC tumor biology. This model closely simulates the clinical features of NPC growth, progression and metastasis and could help elucidate the biological mechanisms of the growth pattern and invasion of NPC cells and in quantitative assessment of angiogenesis and cell intravasation.

## Introduction

Nasopharyngeal carcinoma (NPC) is a unique head and neck cancer with a remarkable geographic and racial distribution worldwide. NPC is rare in most parts of the world but has a high incidence in a few populations such as Southern Chinese (including Hong Kong), South-east Asians, and native Eskimos in Greenland and Alaska [1, 2]. The etiology of NPC is strongly associated with infection with Epstein-Barr virus (EBV) as well as genetic susceptibility and environmental factors [3]. NPC has high invasive and metastatic tendency. NPC patients commonly present skull-base erosion, cranial nerve involvement and cervical lymph node metastasis. Early-stage NPC is curable, but even with concurrent chemoradiotherapy, distant metastasis remains the major cause of treatment failure[4]. Therefore, exploring the molecular basis of NPC invasion and metastasis would greatly help in understanding the biology behavior of NPC and improving NPC therapy.

Despite a number of genetic and epigenetic changes which have been identified in the past however the absence of EBV-genome remains a problem. One of the major obstacles is lack of study materials. The definite diagnosis of NPC is usually confirmed by endoscopic biopsy; thus, the tumor mass is too small for direct study or for xenografts. As open biopsy of cervical lymph nodes increases the possibility of distant metastasis, fine-needle aspiration cytology is the first choice for evaluating suspected cervical lymphadenopathy; thus, the cervical metastatic tumor mass obtained for research is even smaller. Furthermore, radiotherapy is the standard treatment for NPC, which again disallows accessing tumor biopsy tissue after diagnosis. Hence, deciphering the molecular process of nasopharyngeal carcinogenesis and NPC progression from primary tumor tissues is difficult.

Besides lack of NPC tissues, the lack of appropriate *in vitro* and *in vivo* models hampers NPC study. Since 1975, more than 20 NPC cell lines have been derived from NPC tissues. However, most of them, except for C666-1, become EBV-negative after prolonged culture [5]. The physiological relevance of these NPC cell lines, especially EBV-negative lines, is uncertain[5]. It was proposed that three-dimensional cell culture in matrigel might be a sensitive model in simulation of NPC tumorigenesis, however it is still EBV negative [6]. To overcome the limitations of the *in vitro* systems described above, patient-derived xenograft (PDX) lines of NPC, such as C15, C17, C18, X666, NPC/HK2117, NPC/HK1915 and NPC/HK1530 have been developed [7, 8]. PDX models retain the original morphology of human carcinoma cells and most importantly, most of them still harbor the EBV genome. Therefore they provide a more accurate representation of the complex biology of NPC cells. These PDX *in vitro* cancer modeling systems were widely used in the study of NPC pathogenesis and evaluation of new anti-cancer therapeutics [9, 10]. However, the PDX models also have drawbacks, with very low rate of success when using fragments of primary tumor and high cost of mice for tumor propagation. In addition, the integrity of the original neoplasms are not maintained in mice since the human stroma and infiltrating cells are rapidly replaced by a mouse stroma [11].

The chick embryo chorioallantoic membrane (CAM) model has been used as an *in vivo* model for cancer research. The CAM is naturally immunodeficient early during hatching and therefore can tolerate the transplantation of human tumor cells. In this model, tumor cells are inoculated on the chorionic epithelium, for highly visible proliferation and invasion. Furthermore, the model is cost- and time-efficient. Therefore, the CAM model has been used in cancer research such as breast, bladder, prostate, ovarian cancer and head and neck cancers for estimating the dissemination and angiogenesis of cancer cells [12–18]. However, the utilization of the CAM model has not been reported in NPC, although it was once used to evaluate the effect of cyclooxygenase 2 inhibitors on angiogenesis induced by NPC cells [19].

In the present study, we used a CAM system for NPC study. We found 100% tumor formation after inoculating the CAM with NPC cells or primary tumor tissue and addressed the feasibility of applying CAM model to visualize and evaluate tumor growth, invasion and tumor angiogenic activity. The model could be used to quantify intravasated tumor cells, and evaluate the metastatic potential of tumor cells with a great advantage. CAM is a powerful model to study the growth, invasion, angiogenic activity and intravasation of NPC tumor cells.

## Materials and Methods

### Ethics Statement

Ethical permission of this study was granted by the Research Ethics Committee of the First Affiliated Hospital of Guangxi Medical University (Nanning, China).

### Cell Lines and Patients sample processing

Four NPC cell lines were used in the present study, including three EBV-negative cell lines (HONE1, 5-8F and 6-10B)[20, 21], and one EBV-positive cell line (C666-1)[20–23]. Both 5-8F and 6-10B cell lines were generated from NPC cell line SUNE1. They share the same genetic background but differ in biological characters: 5-8F has a high tumorigenic and metastatic ability while 6-10B has a low tumorigenicity and lack of metastatic ability. These cell lines were maintained in Iscove's Modified Dulbecco's Medium (IMDM) (HyClone, USA) supplemented with 10% fetal bovine serum (GIBCO, USA) and 1% penicillin/streptomycin in a humidified atmosphere with 5% CO_2_ at 37°C. GFP-expressing HONE1 (HONE1-GFP) cells were generated by transfecting HONE1 cells with pEGFP-C1 plasmid and were selected by 400 μg/ml geneticin for 2 weeks. Single clones with positive green fluorescent were chosen and maintained in 200 μg/ml geneticin.

A number of 35 NPC primary tumor biopsies were collected from newly diagnosed and untreated NPC patients (aged from 20–66 years, 26 males and 9 females, 33 cases of non-keratinizing carcinoma and 2 cases of keratinizing squamous cell carcinoma) at the department of Otolaryngology-Head and Neck Surgery, First Affiliated Hospital of Guangxi Medical University, with written informed consent from donors. All the NPC patients were diagnosed by experienced pathologists according to the World Health Organization (WHO) classification. Upon collection, tumor tissue samples were briefly stored in IMDM without serum and antibiotics, and carried from the surgery room. Within 30min, the fragments (1.5mm×1.5mm) of tumor were inoculated in CAM models.

### Egg Preparation and Tumor Cell Inoculation

Fertilized chicken eggs were purchased from a local hatchery and incubated for 8 days after breeding at 38°C with 60% humidity. The method for preparing the CAM was described elsewhere with slight modification [14, 15]. In general, eggs were cleaned with pre-warmed 70% ethanol and a small hole was drilled into the eggshell where the air sac is located. A 1-cm^2^ window was carefully opened for inoculated. The small hole was then vacuumed to exclude air, thus creating space for the CAM. A total of 0.5×10^6^ or 1×10^6^ cells resuspended in 20 μl serum and antibiotics free IMDM or NPC primary tumor biopsy tissue was seeded into a silicon ring placed on the CAM. The window was then covered with parafilm and the egg was placed back into the incubator. Silicone rings were removed 24 h later. Tumor growth and embryo viability were examined daily under the SZ61 Zoom Stereo Microscope (Olympus, Japan). Five days after inoculation, micro-tumors were removed from CAMs, paraformaldehyde-fixed and paraffin-embedded. For metastasis study, heart and lung tissue of developing chickens was harvested and stored at -80°C before DNA extraction.

### Analysis of neovascularization ratio

CAMs were carefully removed at the fifth day after inoculation and images were obtained under microscopy immediately. Angiogenesis index is determined using the ImageJ software, by dividing the area of VA (the area of blood vascularization) to the total area of CAM.

### Haematoxylin and Eosin (H&E) Staining and Immunohistochemistry (IHC) Staining Assay

To demonstrate the histological characteristics, H&E staining was carried out following a standard protocol[24]. For immunohistochemistry staining, sections after deparaffinization and rehydration underwent antigen retrieval and blocking, then were incubated with anti-human CK34βE12 antibody (Dako, 1:100) at 4°C overnight, then rabbit anti-mouse secondary antibody with peroxidase-conjugated neutravidin, then visualization by 3,3’-diaminobenzidine (DAB) reagent (ZSGB-BIO). Finally, sections were counterstained with haematoxylin and images were acquired under a microscope (Olympus C-5050, Japan).

### *In Situ* Hybridization (ISH) for Epstein-Barr Virus Encoded Small RNA (EBERs)

To detect the expression of EBV-encoded nuclear RNAs (EBER1 and 2), ISH was performed according to the manufacturer’s instructions (S30172, Triplex Biosciences, China). Briefly, deparaffinized and rehydrated sections were pretreated with proteinase K and hybridized with EBER probe at 55°C for 1.5 h, then washed with pre-warmed phosphate buffered saline solution. Mouse anti-Dig antibody and polymer enhancer were sequentially applied. After incubation with polymerized horseradish peroxidase-conjugated anti-mouse antibody, DAB solution was used for detection. Finally, sections were counterstained with haematoxylin and observed on microscopy.

### Confocal Microscopy

To observe the invasion and metastasis of NPC cells, aliquots of 1×10^6^ HONE1-GFP cells were inoculated on CAMs (n = 5). At 48 h after seeding, the inoculation site and its surrounding CAM were sealed and observed under a confocal microscope and photographed (Nikon A1si, Japan).

### Analysis of Tumor Cells in CAMs by Quantitative PCR (qPCR) Amplification of Human-specific β-Globin Sequence

Briefly, genomic DNA was extracted from 5-8F and 6-10B cells or homogenates of chick heart and lung tissues by use of the TIANamp Genomic DNA kit (TIANGEN, China). The PCR system was previously described [25], with initial denaturation at 95°C for 5 min, followed by 35 cycles of 95°C for 45 sec, 60°C for 45 sec and 72°C for 45 sec, and a final extension at 72°C for 5 min. Primer sequences were for β-globin, sense 5’-GAAGAGCCAAGGACAGGTAC-3’, and antisense 5’-CAACTTCATCCACGTTCACC-3’, generating a product of 264 bp [26, 27]. PCR product was then purified and subcloned into the pMD18-T vector (TAKARA, Japan). Plasmid DNA was quantified spectrophotometrically. A 10-fold serial dilution of the plasmid pMD18-T–β-globin ranging from 5.41×10^11^ to 5.41×10^6^ copies/μl, was used to construct a standard curve. The corresponding copy number of the plasmid was calculated according to the following equation[28]: DNA (copy number) = 6.02×10^23^ (copy/mol) ×DNA amount (g)÷plasmid DNA length (bp)÷660 (g/mol/bp).

We next determined the copy number of human β-globin by qPCR according to the standard curve in a 20-μl reaction system containing 9 μl RealMasterMix (SYBR Green), 1 μl each primer (5 μM), 5 μl genomic DNA and 4 μl distilled water. All samples were subjected to an initial denaturation of 95°C for 3 min, followed by 45 cycles of denaturation at 95°C for 20 sec, 66°C for 20 sec and 72°C for 20 sec. C_T_ values were measured in triplicate and against the standard curve, and the number of tumor cells in each CAM sample was then calculated.

### Statistical Analysis

SPSS 18.0 (SPSS Inc., Chicago, IL) was used for statistical analysis. Student’s *t* test and one-way ANOVA were used to compare data for groups. *P*<0.05 was considered statistically significant.

## Results

### Establishment of NPC Micro-tumors on CAM

**Fig 1:**
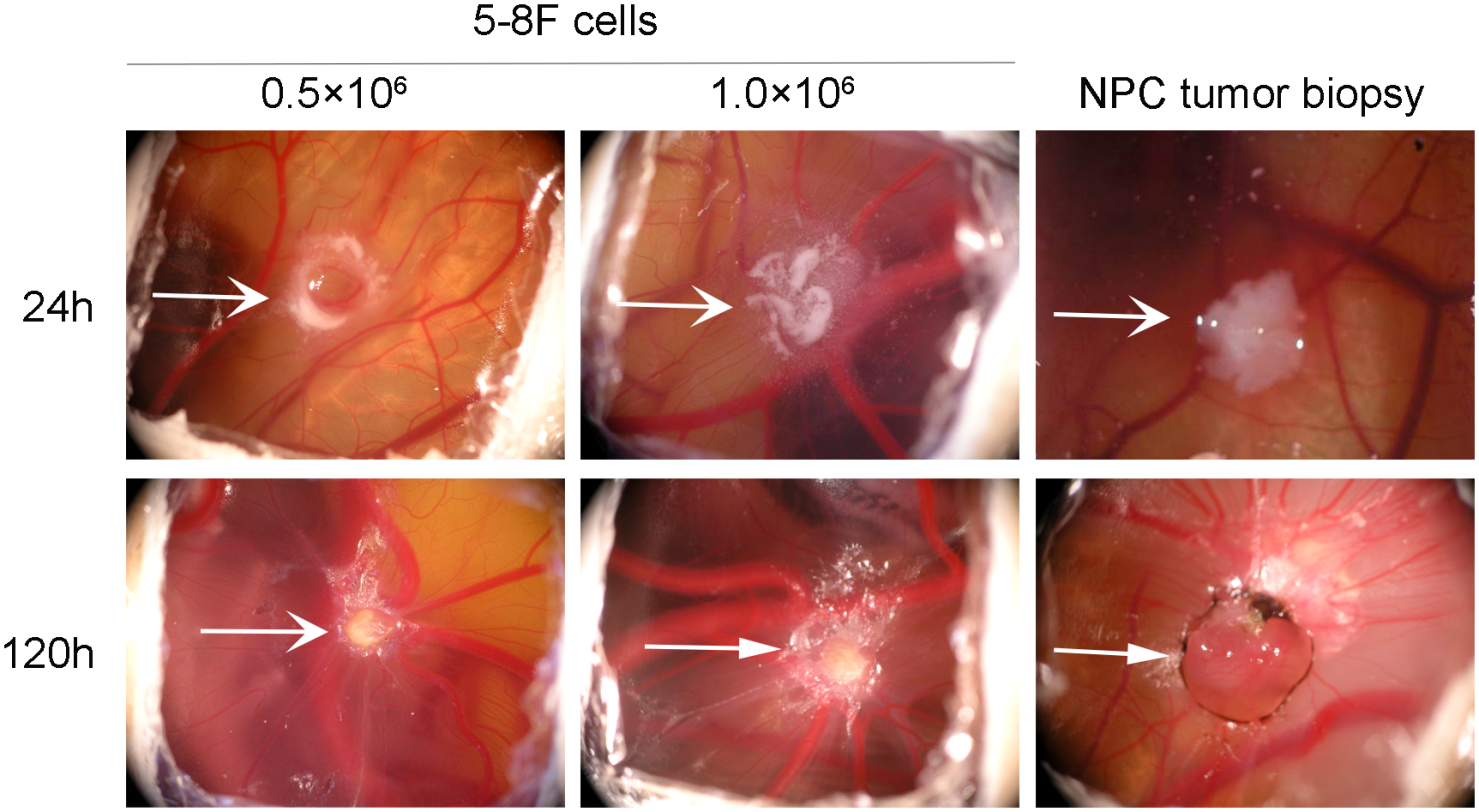
Tumor xenografts in chorioallantoic membranes (CAMs) inoculated with NPC cells or tumor biopsies. CAMs inoculated with 0.5×10^6^ or 1×10^6^ 5-8F cells and NPC primary tumors at 24 and 120 h after inoculation. Representative data are shown.

To access the possibility of using the CAM model to study the tumorigenecity of NPC, we inoculated CAMs with NPC cell lines or primary tumor biopsy tissue. The detailed clinical features of the 35 NPC patients involved in this study were listed in Table 1. Micro-tumor formation and growth were observed and photographed daily. As early as 72 h after inoculation with 0.5×10^6^ NPC cells or NPC tumor biopsy, we found 100% tumor formation (4/4 NPC cell lines and 35/35 NPC tumor biopsies, data not shown). The transplanted xenografts maintained good vitality and growth status before being removed out. Representative data was shown in Fig 1.

**Table 1:** Clinical features of NPC patients participated in this study.

**Case** ^a^	**Age**	**Gender** ^b^	**Histological subtype** ^c^	**Staging** ^d^
T1	65	M	B2	T3N1M0
T2	30	M	B2	T3N2M0
T3	65	M	B2	T3N1M0
T4	42	M	A	T2N1M0
T5	62	M	B2	T3N1M0
T6	37	M	B2	T3N3M0
T7	39	M	B2	T2N1M0
T8	46	M	A	T3N1M0
T9	52	F	B2	T2N0M0
T10	55	F	B2	T3N1M0
T11	45	F	B2	T2N1M0
T12	43	F	B2	T2N1M0
T13	58	M	B2	T2N0M0
T14	52	M	B2	T3N1M0
T15	34	M	B2	T3N2M0
T16	27	M	B2	T3N1M0
T17	64	M	B2	T3N1M0
T18	39	F	B2	T3N0M0
T19	42	M	B2	T2N0M0
T20	66	M	B2	T3N1M0
T21	20	F	B2	T2N1M0
T22	58	F	B2	T3N0M0
T23	53	F	B2	T3N1M0
T24	48	M	B2	T2N1M0
T25	59	M	B2	T3N1M0
T26	34	F	B2	T3N1M0
T27	29	M	B2	T3N1M0
T28	65	M	B2	T2N1M0
T29	61	M	B2	T2N1M0
T30	45	M	B2	T2N1M0
T31	45	M	B2	T3N0M0
T32	60	M	B2	T3N1M0
T33	47	M	B2	T2N1M0
T34	39	M	B2	T2N1M0
T35	36	M	B2	T3N0M0

^a^: NPC patients involved in this study.

^b^: M, male; F, female.

^c^: WHO histopathological classification of NPC (2005), A: keratinizing squamous cell carcinoma; B1: differentiated non-keratinizing carcinoma; B2: undifferentiated non-keratinizing carcinoma; C: Basaloid squamous cell carcinoma.

^d^: The TNM clinical classification for NPC according to AJCC staging, 7th Edition. T-Primary tumor; N-Regional lymph nodes; M-Distant metastasis.

### Histology and Immunohistochemistry Analysis

**Fig 2:**
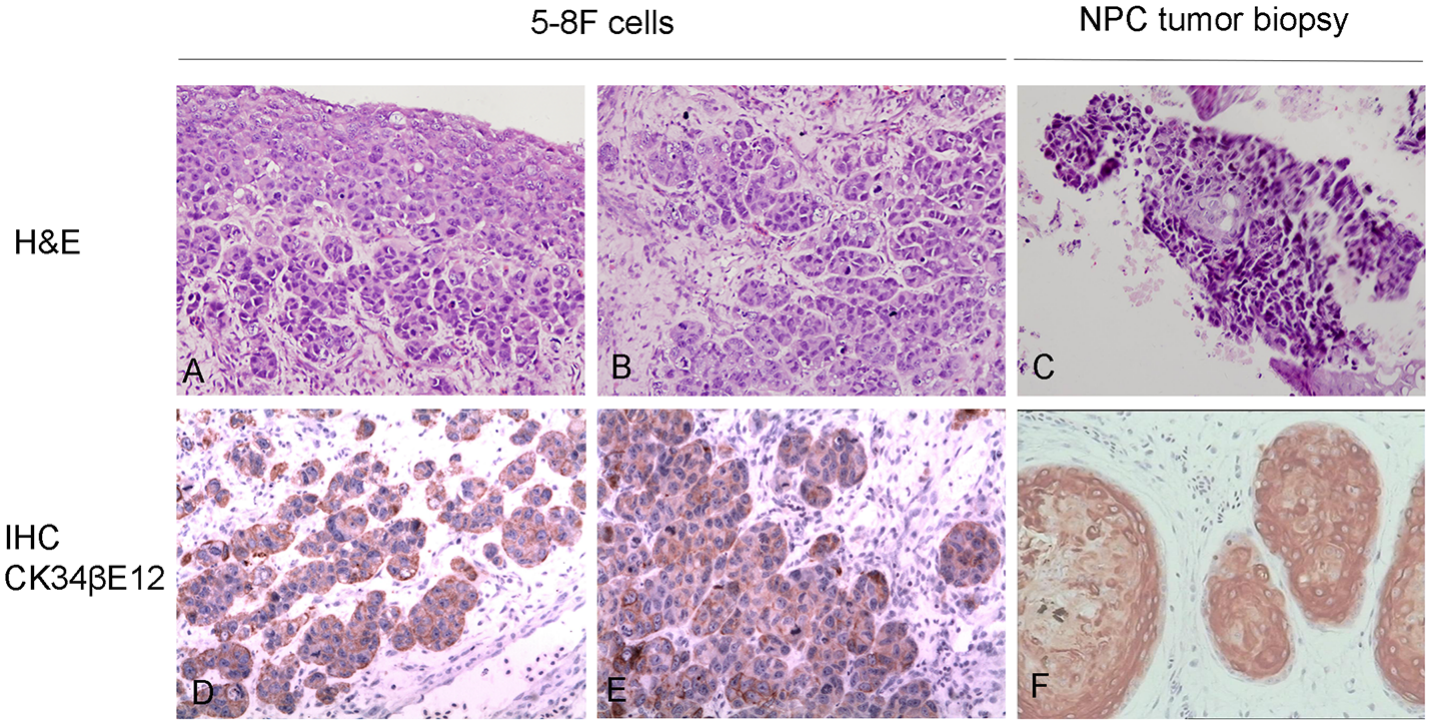
Histochemistry, and immunohistochemistry staining of xenografts. A-C: H&E staining of derived xenografts 120 h after inoculation with 1×10^6^ 5-8F cells or primary tumors. Representative images are shown. D-F: IHC staining of human CK34βE12. Magnification, ×400.

Five days after inoculation with 5-8F cells and NPC tumor biopsy, undifferentiated tumor cells with a homogeneous cell shape and large nuclei were observed in the CAM membrane surface and in the chorioallantoic mesenchymal. In addition, tumor cell nests were present in the membrane stroma (Fig 2A–2C). Immunohistochemistry staining of human cytokeratin CK34βE12 confirmed the epithelial origin of micro-tumors (Fig 2D–2F).

### *In Situ* Hybridization (ISH) of EBERs

**Fig 3:**
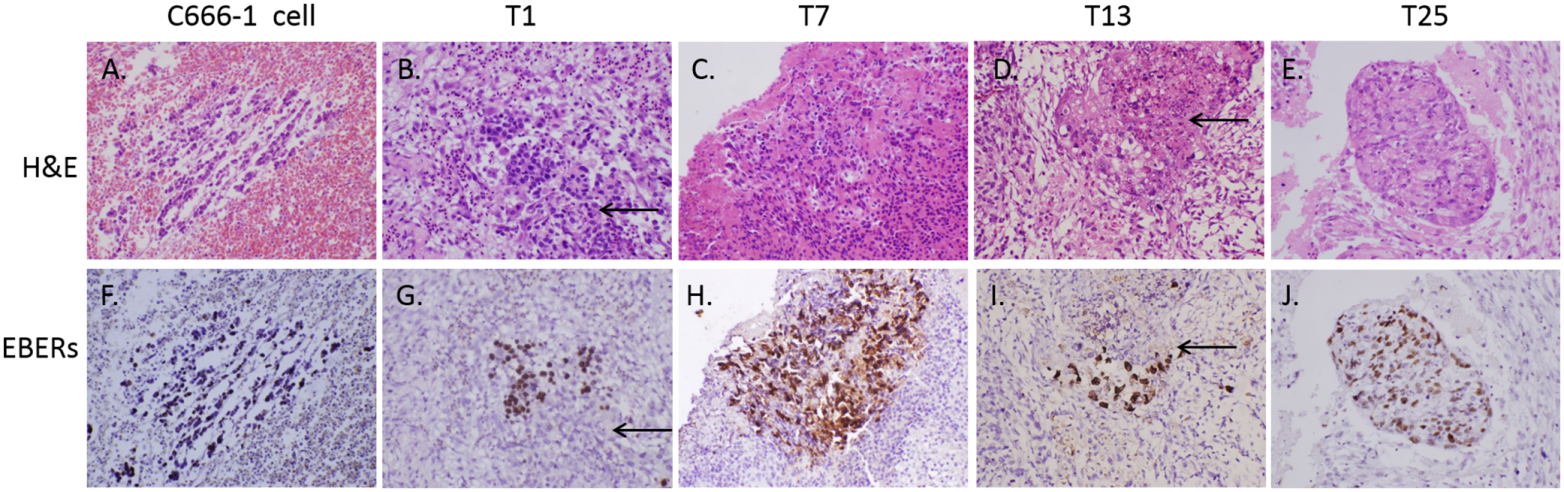
H&E staining and *in situ* hybridization (ISH). H&E staining and *in situ* hybridization detection of Epstein-Barr virus–encoded RNAs (EBERs) on CAMs inoculated with C666-1 cells (A and F) or NPC tumor biopsies (B-E and G-J). Arrows point to tumor areas where malignant NPC cells have become EBER negative. Magnification, ×400.

Considering that NPC is strongly associated with EBV infection, we performed ISH assay in CAMs inoculated with EBV-positive C666-1 cells and all of the 33 undifferentiated NPC tumor biopsies and detected the presence of EBERs in the micro-tumors. As shown in Fig 3, it was noted that EBV could be maintained in CAM model, indicating the possibility of using CAM as a model for studying EBV-related tumor biology. However, EBERs was negative in parts of NPC cells (Fig 3B, 3D, 3G and 3I), presumably due to the rapid switching of microenvironment leading to tumor necrosis and/or EBV lost. Further studies are needed to elucidate this phenomenon.

Histologically, the transplanted NPC tumors retained poor differentiation. Most of the morphology of NPC tumor cells was retained, indicating that the inoculated NPC biopsies were surviving in CAMs. Besides, a loose connective tissue including spindle fibroblasts and collagen fibers possibly derived from chick embryos surrounding the NPC tissues. Lymphocytes and scant neutrophils were also observed.

### Tumor Invasion and Metastasis in CAM

**Fig 4:**
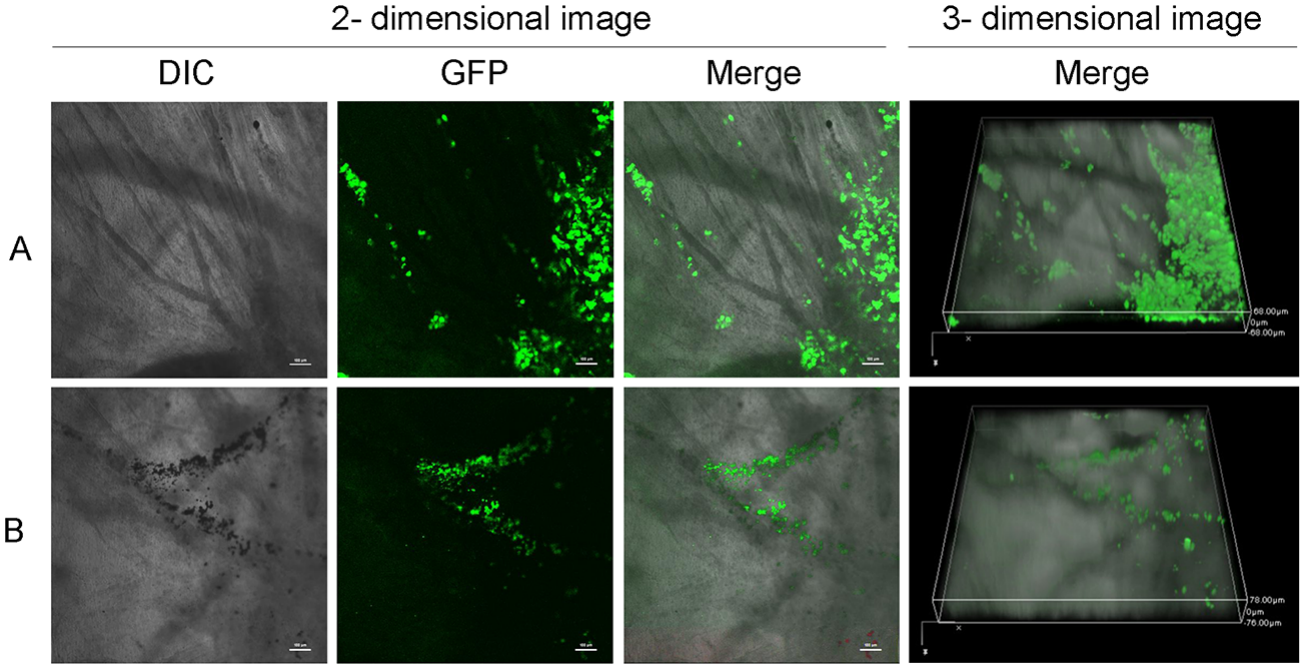
2D and 3D analysis of invasion and metastasis of NPC cells 48 h after inoculation. A: Invasion of HONE1-GFP cells through the basement membrane and toward the lower site of CAM, especially in 3D imaging. Invasive depth is shown in the Z-axis. B: Metastasis of HONE1-GFP cells via the blood system. Representative bright-field (DIC), green fluorescent (GFP) and overlay (Merge) images. Magnification, ×40.

We next evaluated the feasibility of visualizing the invasion and metastasis of NPC cells in the CAM model. At 48 h after inoculation with HONE1-GFP cells, CAMs were carefully removed and observed by confocal microscopy. Blood vessels were visualized on bright-field microscopy and HONE1-GFP cells by fluorescence (Fig 4). Intravasated tumor cells were disseminated along large vessels. The growth and invasion of HONE1-GFP cells was clear, and the invasion depth of micro-tumors could be accessed in a 3D pattern.

### Evaluation of the Angiogenetic Activity of NPC Cell Lines on CAM

**Fig 5:**
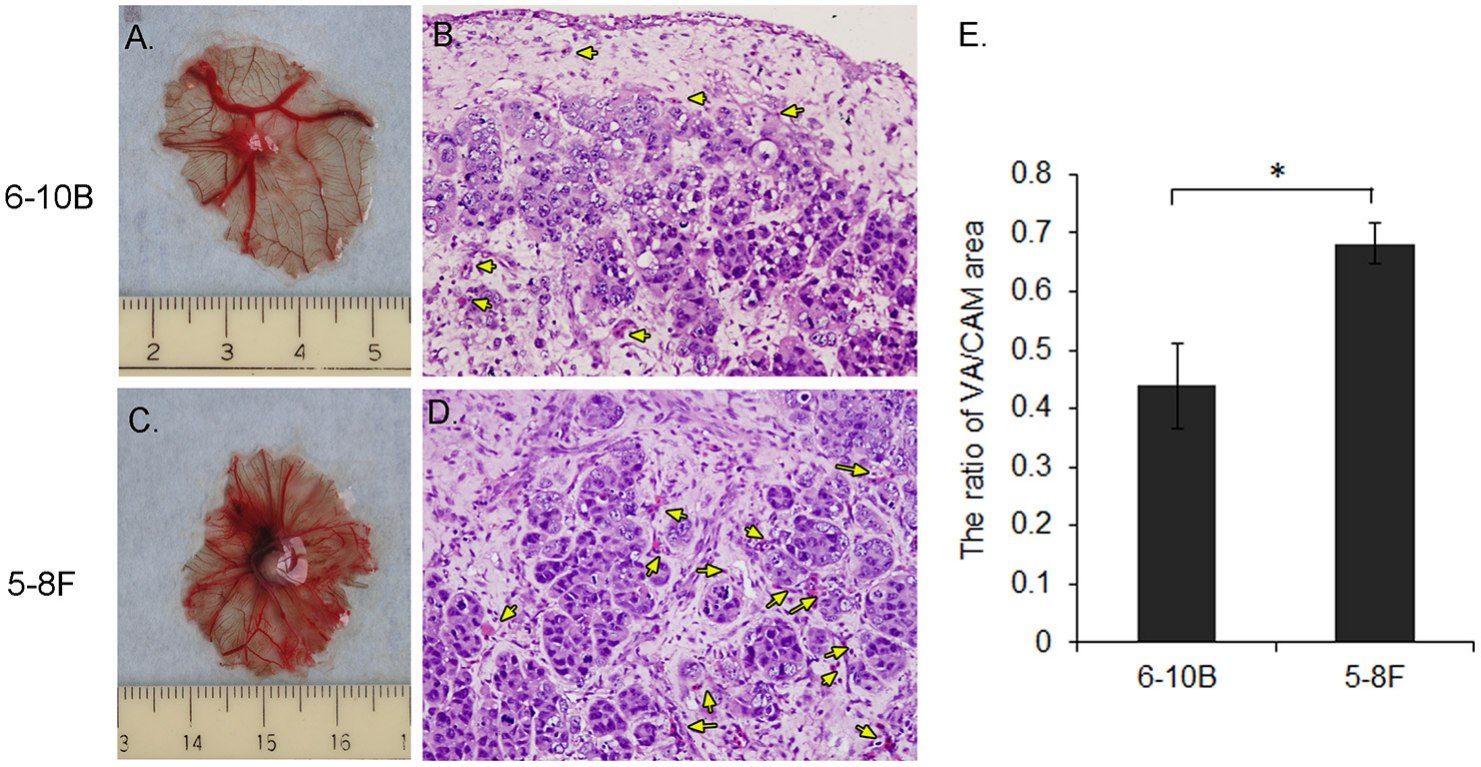
Neovascularization in CAMs after inoculation with 5-8F and 6-10B cells. A and C: Macro images of xenografts and blood vessels. B and D: H&E staining. Blood vessels with nucleated erythrocytes are indicated with yellow arrows. E. Quantification of neovascularization ratio calculated by dividing the area of blood vascularization (VA) to total area of CAM. Data are mean ± SD (n = 3). * *P*<0.01.

Besides having invasion and metastasis ability, NPC cells show significant angiogenetic activity on CAMs. To further evaluate this, we inoculated CAMs with 2 NPC cell lines, 5-8F (high tumorigenic and metastatic) and 6-10B (low tumorigenic and metastatic) [29], to compare their angiogenesis capability. Both of the two NPC cell lines induced neo-angiogenesis shown in Fig 5. As expected, 5-8F cells greatly induced new blood vessels (Fig 5A and 5C), which was further supported by H&E staining (Fig 5B and 5D, yellow arrows). In quantifying the angiogenesis potential of the two cell lines, the ratio of vascularization to total CAM area for 5-8F was greater than 6-10B cells, up to 1.5-fold (*P* = 0.008, Fig 5E). Thus, CAM could be an appropriate model for NPC angiogenesis research.

### Metastatic Potential of NPC cells in CAMs

**Fig 6:**
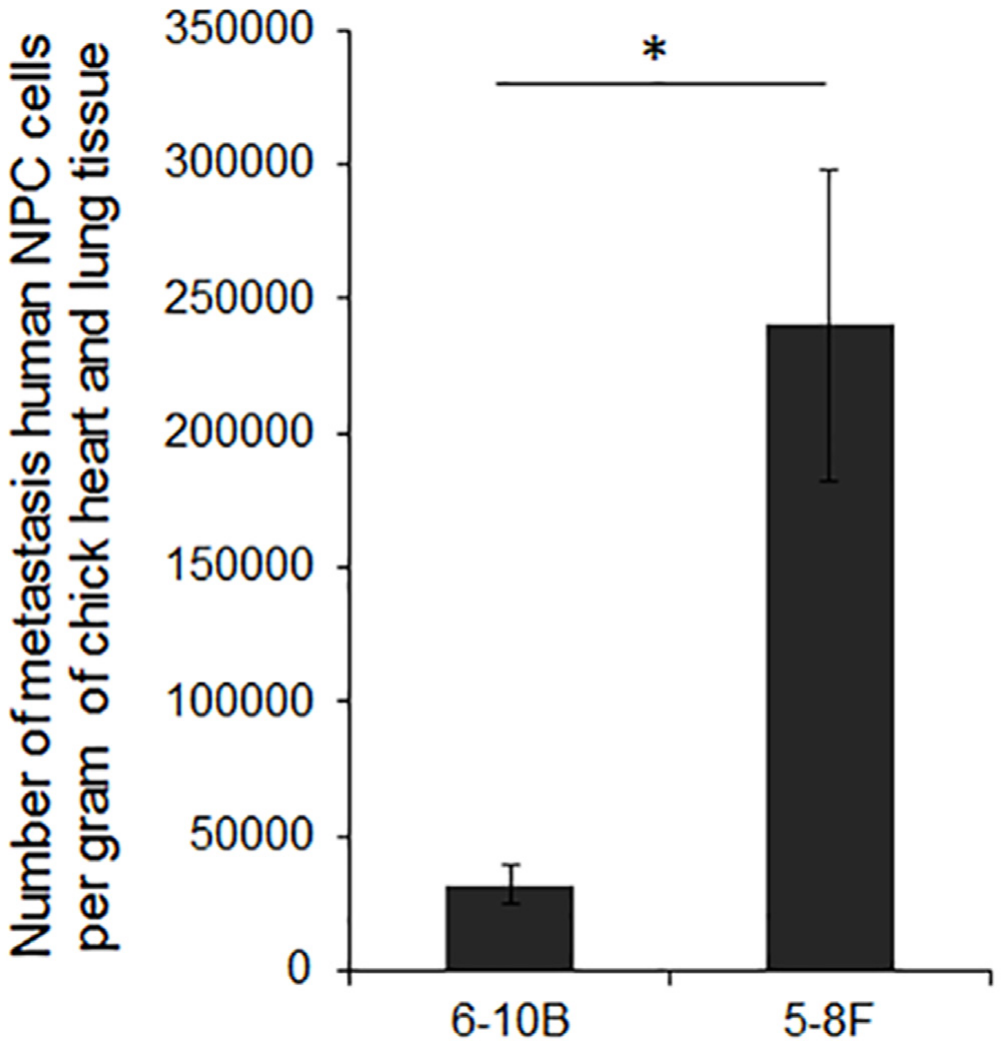
Quantification of disseminating NPC cells in CAM. Number of circulating 5-8F and 6-10B cells in the lung and heart of developing chicken evaluated by β-globin–based qPCR and expressed as mean ± SD (n = 5 for each cell line). * *P*<0.05.

To further quantify the metastatic ability of 5-8F and 6-10B cells inoculated on CAMs, we performed qPCR amplification of the human-specific β-globin gene. At 120 h after inoculation, the number of cells was higher for 5-8F than 6-10B cells in the lung and heart of chicks with inoculated CAMs, which indicates stronger intravasation and metastasis ability for 5-8F than 6-10B cells (*P*<0.05, Fig 6). This result is consistent with the cellular phenotypes of 5-8F and 6-10B cells. Hence, CAM is a feasible model for quantitative analysis of the metastatic ability of NPC cells.

## Discussion

NPC is a highly invasive and highly metastatic head and neck cancer. However, mechanistic study of the invasion and metastasis of NPC has been hampered by the limitation of appropriate *in vivo* models. We established an *in vivo* chick embryo CAM model to study NPC tumor biology and found 100% NPC micro-tumor formation 3 days after inoculation with NPC cell lines or primary tumor biopsy tissue. The transplanted NPC micro-tumors grew on CAM with extracellular matrix (ECM) interaction and induced angiogenesis. This feasible, easy-to-manipulate and reliable CAM model could be used to study the growth of transplanted NPC tumors and several important steps of metastasis.

An appropriate tumor model is important and necessary for studying NPC tumor progression *in vivo*. To date, the *in vivo* NPC models are mainly established by ectopic subcutaneous implantation of human NPC tissue in immunodeficient mice. For example, C15 was derived from a primary NPC tumor, while C17 and C18 were derived from metastatic NPC tissue, being propagated in Swiss nude. Crucially, these models retain EBV DNA and express EBV latent proteins[8]. Since most of the established NPC cell lines lost EBV after passages *in vitro*, these EBV-positive PDX lines provide more valuable information to study the etiological role of EBV in epithelial cells and the contribution of EBV to NPC development, thus have been widely used in various groups worldwide [10, 30–33]. However, transplantation of primary tumor biopsy tissue into nude mice has not been efficacious, and maintaining the tumor-bearing nude mice models is time-consuming. Moreover, it is difficult to observe the xeno-transplanted tumor growth dynamically.

The CAM model has many advantages for tumorigenesis study: due to the lack of B- and T-cell mediated immune function early during hatching, the chick embryo system is naturally immunodeficient and may accept xeno-transplantation from human tumors. We established the CAM, as an *in vivo* model for short-term study of NPC tumor growth and metastasis. With xeno-transplanting micro-tumors on CAM, we can study the growth of transplanted NPC tumors and several critical steps of metastasis: tumor invasion ability by detecting the extent of basement membrane penetration, tumor angiogenic activity by analyzing the area of neo-vessels, and tumor metastasis by quantifying tumor cells in distant organs, such as lung and heart. As well, these analyses can be completed in the same model within 5 days, with very low cost.

The CAM model of NPC demonstrated excellent tumor formation, 100% for all 4 NPC cell lines and NPC biopsies used. Tumor cell clusters grew rapidly and formed 3D micro-tumors within 5 days after inoculation. These NPC micro-tumors showed interaction with the ECM, strongly induced angiogenic activity in the host membrane and had high potential to invade the chorioallantoic membrane.

Much experimental evidence indicates that tumor growth depends on angiogenesis. Moreover, the extent of vascularization is a key predictor for human NPC metastatic risk. Increased microvessel count was associated with progression of regional lymph node involvement in NPC patients [34]. However, most of the studies used metastasized organs as endpoints. Intravasation, as the most important step in the metastasis process, is not well investigated. The major obstacle is the inefficiency of this process, leading to a difficulty of tracking the tumor cells movement *in vivo*. PCR-based assay depending on tissue-specific genes such as cytokeratins is a highly sensitive method for detecting circulating NPC cells. However, this method has only been attempted with NPC patients and demonstrated no statistical significance[35]. We lack an *in vivo* NPC model that can be used to study the dynamic process from invasion to intravasation. Using GFP-expressing NPC cells, we established fluorescent NPC micro-tumors in the CAM model, thus enabling the imaging of growth, invasion and intravasation of NPC cells. Host vessels induced around the growing tumor could be distinguished clearly against the background of a brilliant fluorescent tumor. Neovessel development and intravasated NPC cells could be observed in real time and quantified, thereby demonstrating the relationship of angiogenesis and intravasation in NPC cancer progression.

Using two NPC cell lines with different metastatic potential (5-8F and 6-10B cells), we observed a significant difference in intravasated cell numbers in the lung and heart of CAM models. These data are consistent with the phenotype of the 2 cell lines and in good agreement with previous *in vitro* and *in vivo* studies. These data demonstrate the feasibility of investigating the multistep metastasis process *in vivo* by a CAM model. Thus, this model should be of importance in understanding the biology of NPC metastasis.

In conclusion, we established and described a feasible, easy-to-manipulate and reliable CAM model for *in vivo* NPC study. This model closely simulates the clinical features of NPC growth, progression and metastasis. The CAM model of NPC could play a critical role in elucidating the tumor biological mechanisms involved in the growth pattern and invasion of NPC cells as well as quantitative assessment of angiogenesis and cell intravasation. In addition, it has high value in the pharmacological and translational field of NPC.

## References

[1] Chan AT, Teo PM, Johnson PJ. Nasopharyngeal carcinoma. Annals of oncology: official journal of the European Society for Medical Oncology / ESMO. 2002;13(7):1007–15. . 12176778 10.1093/annonc/mdf179

[2] Adham M, Greijer AE, Verkuijlen SA, Juwana H, Fleig S, Rachmadi L, et al Epstein-Barr virus DNA load in nasopharyngeal brushings and whole blood in nasopharyngeal carcinoma patients before and after treatment. Clinical cancer research: an official journal of the American Association for Cancer Research. 2013;19(8):2175–86. 10.1158/1078-0432.CCR-12-2897 . 23493345

[3] Chang ET, Adami HO. The enigmatic epidemiology of nasopharyngeal carcinoma. Cancer epidemiology, biomarkers & prevention: a publication of the American Association for Cancer Research, cosponsored by the American Society of Preventive Oncology. 2006;15(10):1765–77. 10.1158/1055-9965.EPI-06-0353 . 17035381

[4] Wei WI, Sham JS. Nasopharyngeal carcinoma. Lancet. 2005;365(9476):2041–54. 10.1016/S0140-6736(05)66698-6 . 15950718

[5] Gullo C, Low WK, Teoh G. Association of Epstein-Barr virus with nasopharyngeal carcinoma and current status of development of cancer-derived cell lines. Annals of the Academy of Medicine, Singapore. 2008;37(9):769–77. . 18989494

[6] Wan XB, Fan XJ, Chen MY, Xu J, Long ZJ, Hua YJ, et al Inhibition of Aurora-A results in increased cell death in 3-dimensional culture microenvironment, reduced migration and is associated with enhanced radiosensitivity in human nasopharyngeal carcinoma. Cancer biology & therapy. 2009;8(15):1500–6. . 19502819 10.4161/cbt.8.15.8958

[7] Huang DP, Ho JH, Chan WK, Lau WH, Lui M. Cytogenetics of undifferentiated nasopharyngeal carcinoma xenografts from southern Chinese. Int J Cancer. 1989;43(5):936–9. Epub 1989/05/15. . 2714899 10.1002/ijc.2910430535

[8] Busson P, Ganem G, Flores P, Mugneret F, Clausse B, Caillou B, et al Establishment and characterization of three transplantable EBV-containing nasopharyngeal carcinomas. International journal of cancer Journal international du cancer. 1988;42(4):599–606. . 2971626 10.1002/ijc.2910420422

[9] Cheung CC, Chung GT, Lun SW, To KF, Choy KW, Lau KM, et al miR-31 is consistently inactivated in EBV-associated nasopharyngeal carcinoma and contributes to its tumorigenesis. Mol Cancer. 2014;13:184 Epub 2014/08/08. 10.1186/1476-4598-13-184 25098679 PMC4127521

[10] Gressette M, Verillaud B, Jimenez-Pailhes AS, Lelievre H, Lo KW, Ferrand FR, et al Treatment of nasopharyngeal carcinoma cells with the histone-deacetylase inhibitor abexinostat: cooperative effects with cis-platin and radiotherapy on patient-derived xenografts. PLoS One. 2014;9(3):e91325 Epub 2014/03/13. 10.1371/journal.pone.0091325 24618637 PMC3949989

[11] Giovanella BC, Stehlin JS, Williams LJ Jr. Heterotransplantation of human malignant tumors in "nude" thymusless mice. II. Malignant tumors induced by injection of cell cultures derived from human solid tumors. Journal of the National Cancer Institute. 1974;52(3):921–30. . 4524119 10.1093/jnci/52.3.921

[12] Mira E, Lacalle RA, Gomez-Mouton C, Leonardo E, Manes S. Quantitative determination of tumor cell intravasation in a real-time polymerase chain reaction-based assay. Clinical & experimental metastasis. 2002;19(4):313–8. . 12090471 10.1023/a:1015563031769

[13] Busch C, Krochmann J, Drews U. The chick embryo as an experimental system for melanoma cell invasion. PloS one. 2013;8(1):e53970 10.1371/journal.pone.0053970 23342051 PMC3544663

[14] Kunzi-Rapp K, Genze F, Kufer R, Reich E, Hautmann RE, Gschwend JE. Chorioallantoic membrane assay: vascularized 3-dimensional cell culture system for human prostate cancer cells as an animal substitute model. J Urol. 2001;166(4):1502–7. . 11547121 10.1016/s0022-5347(05)65820-x

[15] Liu M, Scanlon CS, Banerjee R, Russo N, Inglehart RC, Willis AL, et al The Histone Methyltransferase EZH2 Mediates Tumor Progression on the Chick Chorioallantoic Membrane Assay, a Novel Model of Head and Neck Squamous Cell Carcinoma. Transl Oncol. 2013;6(3):273–81. 23730406 10.1593/tlo.13175 PMC3660795

[16] Ribatti D, Vacca A, Roncali L, Dammacco F. The chick embryo chorioallantoic membrane as a model for in vivo research on angiogenesis. The International journal of developmental biology. 1996;40(6):1189–97 9032025

[17] Lokman NA, Elder AS, Ricciardelli C, Oehler MK. Chick Chorioallantoic Membrane (CAM) Assay as an In Vivo Model to Study the Effect of Newly Identified Molecules on Ovarian Cancer Invasion and Metastasis. International journal of molecular sciences. 2012;13(8):9959–70. 10.3390/ijms13089959 22949841 PMC3431839

[18] Lester RD, Jo M, Montel V, Takimoto S, Gonias SL. uPAR induces epithelial-mesenchymal transition in hypoxic breast cancer cells. The Journal of cell biology. 2007;178(3):425–36. 10.1083/jcb.200701092 17664334 PMC2064849

[19] Chen PY, Long QC. Effects of cyclooxygenase 2 inhibitors on biological traits of nasopharyngeal carcinoma cells. Acta pharmacologica Sinica. 2004;25(7):943–9. . 15210070

[20] Yao KT, Zhang HY, Zhu HC, Wang FX, Li GY, Wen DS, et al Establishment and characterization of two epithelial tumor cell lines (HNE-1 and HONE-1) latently infected with Epstein-Barr virus and derived from nasopharyngeal carcinomas. International journal of cancer Journal international du cancer. 1990;45(1):83–9. . 2153642 10.1002/ijc.2910450116

[21] Song LB, Yan J, Jian SW, Zhang L, Li MZ, Li D, et al [Molecular mechanisms of tumorgenesis and metastasis in nasopharyngeal carcinoma cell sublines]. Ai zheng = Aizheng = Chinese journal of cancer. 2002;21(2):158–62. . 12479066

[22] Cheung ST, Huang DP, Hui AB, Lo KW, Ko CW, Tsang YS, et al Nasopharyngeal carcinoma cell line (C666-1) consistently harbouring Epstein-Barr virus. International journal of cancer Journal international du cancer. 1999;83(1):121–6. . 10449618 10.1002/(sici)1097-0215(19990924)83:1<121::aid-ijc21>3.0.co;2-f

[23] Chan SY, Choy KW, Tsao SW, Tao Q, Tang T, Chung GT, et al Authentication of nasopharyngeal carcinoma tumor lines. International journal of cancer Journal international du cancer. 2008;122(9):2169–71. 10.1002/ijc.23374 . 18196576

[24] Fischer AH, Jacobson KA, Rose J, Zeller R. Hematoxylin and eosin staining of tissue and cell sections. CSH protocols. 2008;2008:pdb prot4986. 10.1101/pdb.prot4986 . 21356829

[25] Zhang Z, Sun D, Van do N, Tang A, Hu L, Huang G. Inactivation of RASSF2A by promoter methylation correlates with lymph node metastasis in nasopharyngeal carcinoma. International journal of cancer Journal international du cancer. 2007;120(1):32–8. 10.1002/ijc.22185 . 17013896

[26] Avissar P, Malinowski DP. Detection and genotyping analysis of human papillomavirus isolates from liquid-based cervical cytology specimens. Methods in molecular biology. 2009;511:361–70. 10.1007/978-1-59745-447-6_16 . 19347306

[27] Garcia-Closas M, Egan KM, Abruzzo J, Newcomb PA, Titus-Ernstoff L, Franklin T, et al Collection of genomic DNA from adults in epidemiological studies by buccal cytobrush and mouthwash. Cancer epidemiology, biomarkers & prevention: a publication of the American Association for Cancer Research, cosponsored by the American Society of Preventive Oncology. 2001;10(6):687–96. . 11401920

[28] Whelan JA, Russell NB, Whelan MA. A method for the absolute quantification of cDNA using real-time PCR. Journal of immunological methods. 2003;278(1–2):261–9. . 12957413 10.1016/s0022-1759(03)00223-0

[29] Liu T, Ding Y, Xie W, Li Z, Bai X, Li X, et al An imageable metastatic treatment model of nasopharyngeal carcinoma. Clinical cancer research: an official journal of the American Association for Cancer Research. 2007;13(13):3960–7. 10.1158/1078-0432.CCR-07-0089 . 17606730

[30] Zhang Z, Sun D, Hutajulu SH, Nawaz I, Nguyen Van D, Huang G, et al Development of a Non-Invasive Method, Multiplex Methylation Specific PCR (MMSP), for Early Diagnosis of Nasopharyngeal Carcinoma. PLoS One. 2012;7(11):e45908 Epub 2012/11/13. 10.1371/journal.pone.0045908 23144779 PMC3489875

[31] Sun D, Zhang Z, Van do N, Huang G, Ernberg I, Hu L. Aberrant methylation of CDH13 gene in nasopharyngeal carcinoma could serve as a potential diagnostic biomarker. Oral oncology. 2007;43(1):82–7. 10.1016/j.oraloncology.2006.01.007 . 16807071

[32] Gilligan KJ, Rajadurai P, Lin JC, Busson P, Abdel-Hamid M, Prasad U, et al Expression of the Epstein-Barr virus BamHI A fragment in nasopharyngeal carcinoma: evidence for a viral protein expressed in vivo. Journal of virology. 1991;65(11):6252–9. 1656092 10.1128/jvi.65.11.6252-6259.1991 PMC250325

[33] Brooks L, Yao QY, Rickinson AB, Young LS. Epstein-Barr virus latent gene transcription in nasopharyngeal carcinoma cells: coexpression of EBNA1, LMP1, and LMP2 transcripts. Journal of virology. 1992;66(5):2689–97. 1313894 10.1128/jvi.66.5.2689-2697.1992 PMC241023

[34] Wakisaka N, Wen QH, Yoshizaki T, Nishimura T, Furukawa M, Kawahara E, et al Association of vascular endothelial growth factor expression with angiogenesis and lymph node metastasis in nasopharyngeal carcinoma. The Laryngoscope. 1999;109(5):810–4. . 10334236 10.1097/00005537-199905000-00024

[35] Lin JC, Tsai CS, Wang WY, Jan JS. Detection of circulating tumor cells in venous blood of nasopharyngeal carcinoma patients by nested reverse transcriptase-polymerase chain reaction. The Kaohsiung journal of medical sciences. 2000;16(1):1–8. . 10741009

